# Sporadic Creutzfeldt-Jakob disease in adults over 80 years: a 10-year review of United Kingdom surveillance

**DOI:** 10.1093/ageing/afae086

**Published:** 2024-05-05

**Authors:** Eugene Ace McDermott, Neil Watson, Johnny Tam, John Centola, Hatice Kurucu King, Jan Mackenzie, David Summers, Alison Green, Marcelo A Barria, Colin Smith, Suvankar Pal

**Affiliations:** The National CJD Research & Surveillance Unit, Centre for Clinical Brain Sciences, University of Edinburgh, Edinburgh, UK; The National CJD Research & Surveillance Unit, Centre for Clinical Brain Sciences, University of Edinburgh, Edinburgh, UK; The National CJD Research & Surveillance Unit, Centre for Clinical Brain Sciences, University of Edinburgh, Edinburgh, UK; The National CJD Research & Surveillance Unit, Centre for Clinical Brain Sciences, University of Edinburgh, Edinburgh, UK; The National CJD Research & Surveillance Unit, Centre for Clinical Brain Sciences, University of Edinburgh, Edinburgh, UK; The National CJD Research & Surveillance Unit, Centre for Clinical Brain Sciences, University of Edinburgh, Edinburgh, UK; The National CJD Research & Surveillance Unit, Centre for Clinical Brain Sciences, University of Edinburgh, Edinburgh, UK; The National CJD Research & Surveillance Unit, Centre for Clinical Brain Sciences, University of Edinburgh, Edinburgh, UK; The National CJD Research & Surveillance Unit, Centre for Clinical Brain Sciences, University of Edinburgh, Edinburgh, UK; The National CJD Research & Surveillance Unit, Centre for Clinical Brain Sciences, University of Edinburgh, Edinburgh, UK; The National CJD Research & Surveillance Unit, Centre for Clinical Brain Sciences, University of Edinburgh, Edinburgh, UK

**Keywords:** Creutzfeldt-Jakob disease, prion, late-onset, older people, phenotype

## Abstract

**Introduction:**

Sporadic Creutzfeldt-Jakob disease (sCJD) is a rapidly progressive neurodegenerative disease with public health implications. Mean age of onset is 68 years. Age-specific incidence declines after 80 years. This may arise from under-ascertainment or other biological features of the disease. Accurate characterisation of late-onset sCJD is important for early diagnosis, avoiding unnecessary investigations and improving ascertainment for public health purposes.

**Objective:**

To phenotype the clinical features and investigation profile of sCJD in adults >80 years.

**Methods:**

We analysed all probable and definite sCJD cases identified by the UK National CJD Research & Surveillance Unit over a 10-year period (2011–2021). Individuals were grouped by age of onset. Clinical features and investigation profiles were compared.

**Results:**

10.3% (123/1196) had an age of onset over 80. Median survival was shorter (3.2 vs 4.3 months; *P* < 0.001). Pyramidal signs (48.3% vs 34.2%; *P* = 0.008) and akinetic mutism (55.1% vs 33.2%; *P* < 0.001) were more frequent. Psychiatric symptoms (26.3% vs 39.6%; *P* = 0.01) and cerebellar signs (65.4% vs 78.6%, *P* = 0.007) were less frequent. Cognitive impairment and myoclonus were highly prevalent regardless of age. Between age groups, the diagnostic sensitivity of cerebrospinal fluid real-time quaking-induced conversion (CSF RT-QuIC) (92.9% vs 91.9%, *P* = 0.74) was comparable, electroencephalography was superior (41.5% vs 25.4%; *P* = 0.006) and MRI was inferior (67.8% vs 91.4%; *P* < 0.001).

**Conclusions:**

Late-onset sCJD has distinct clinical features, shorter survival and a different profile of investigation sensitivity. CSF RT-QuIC, MRI brain and specialist CJD review is recommended in older adults with a rapidly progressive neurological disorder. Autopsy is valuable when the cause remains elusive.

## Key Points

- This population-based surveillance study characterises the clinical phenotype and investigation profile in sporadic Creutzfeldt-Jakob disease (sCJD) in adults over 80.
- Older adults with sCJD demonstrate a rapid course with a shorter disease duration.
- MRI brain alone may not identify cases as readily due to its lower sensitivity in older adults.
- CSF RT-QuIC is underutilised in older adults, despite a sensitivity of 92.9%.
- In suspected cases, we recommend CSF RT-QuIC, MRI brain and referral to a specialist prion unit.

## Background

Human prion diseases are rapidly progressive and uniformly fatal neurodegenerative disorders associated with significant public health risks due to their transmissible nature. Creutzfeldt-Jakob disease (CJD) surveillance programmes are active globally to monitor epidemiological trends and mitigate these risks. There are sporadic, genetic and acquired forms of disease, with sporadic Creutzfeldt-Jakob disease (sCJD) accounting for the majority (85–90%) of individuals [1, 2]. Typically, sCJD presents as a rapidly progressive dementia with associated clinical features. These include pyramidal, extrapyramidal and cerebellar signs, cortical visual impairment, myoclonus and ultimately akinetic mutism. Clinical phenotypes are heterogeneous, and are influenced by the polymorphism at codon 129 of the prion protein gene (*PRNP*) and the prion protein isotype. The combination informs the molecular classification in sCJD via the system proposed by Parchi *et al.* [3].

The annual incidence and mortality rate of sCJD is reported as 1–2 per million [2, 4, 5]. In the UK, the incidence has been steadily increasing [5]. Proposed explanations include increased vigilance following the emergence of variant CJD, improvements in diagnostics and updated diagnostic criteria with improved sensitivity [5–8]. Age-specific incidence increases with advancing age in Alzheimer’s disease and Parkinson’s disease [9, 10]. In sCJD, this declines over the age of 80 [5, 11, 12]. A similar decline is observed in motor neuron disease and frontotemporal lobar degeneration syndromes [13, 14]. Explanations may include under-ascertainment in older individuals and protective biological factors. Missed cases may be due to atypical clinical presentations, misattribution of clinical features to other comorbidities and neurodegenerative disorders, and lack of access to appropriate investigations. Furthermore, in suspected sCJD, it is feasible that older age may be associated with atypical performance of diagnostic investigations.

In the UK, the National CJD Research & Surveillance Unit (NCJDRSU) provides comprehensive case ascertainment of all forms of human prion disease [15]. The unit assesses referrals from neurologists, geriatricians and psychiatrists in both inpatient and community settings. Individuals with suspected sCJD undergo dedicated clinical assessment. The unit provides expertise in neuroimaging interpretation, cerebrospinal fluid (CSF) biomarkers and neuropathological examination. Diagnosis is challenging, and for rapidly progressive dementia, the differential diagnosis is broad, including important treatable and reversible diagnoses [16, 17]. Consensus diagnostic criteria include evaluation of cardinal clinical features, magnetic resonance imaging (MRI) brain, CSF biomarkers and electroencephalography (EEG) [18]. Neuropathological examination can achieve definitive diagnosis, although the sensitivity of CSF real-time quaking-induced conversion (RT-QuIC) and MRI are such that autopsies are now infrequently pursued [5].

Older individuals with sCJD have not been characterised definitively from a population-based cohort since the implementation of new diagnostic criteria incorporating CSF RT-QuIC and cortical ribboning changes on MRI brain in 2017. A recent study from our group reported on particular clinical features, prolonged survival and lower RT-QuIC sensitivity in sCJD <50 years [19]. Accurate characterisation of polar extremes of age may support improved diagnosis, accurate case ascertainment for public health purposes and avoidance of unnecessary investigations in this group. With an ageing population, there is an unmet need for evaluation of older individuals with sCJD to better understand clinical phenotypes, performance of diagnostic investigations, neuropathological features and overall performance of existing diagnostic criteria.

### Aims and objectives

We aimed to characterise late-onset sCJD (aged ≥80) by evaluating clinical features and performance of diagnostic investigations.

### Hypotheses

We hypothesised that in late-onset sCJD:

1. Individuals may present with atypical clinical phenotypes.
2. Survival may be shorter (due to comorbidities and lack of biological reserve).
3. Diagnostic test sensitivity may differ from that of younger individuals.
4. Individuals may be more frequently diagnosed at autopsy rather than in-life due to lack of ascertainment/early diagnosis.

## Methods

### Study design

We evaluated prospective UK CJD surveillance data from the NCJDRSU. Our protocol details the case-ascertainment process [17].

### Study population

We identified all cases of sCJD referred to the NCJDRSU between 31 August 2011 and 31 August 2021. We included cases of probable or definite sCJD using the 2017 CJD International Surveillance Network diagnostic criteria [15]. Neuropathological confirmation is required for classification as definite sCJD. Sufficient clinical features with an indicative investigation finding (MRI, EEG or CSF biomarker) are required for classification as probable sCJD. The 2017 criteria were applied to referrals seen before 2017. We defined late-onset sCJD as an age of onset of ≥80 years.

### Clinical data

Clinical data were obtained from the NCJDRSU database. All individuals with suspected sCJD undergo comprehensive structured assessments by NCJDRSU neurologists. This includes patient and family interview, physical examination and review of medical notes. Assessments are performed either in-person or remotely via telehealth [20]. We defined disease duration as the time from symptom onset to death, time-to-diagnosis as the time from symptom onset to diagnosis, and date of diagnosis as the date an individual received a diagnosis of probable or definite sCJD. We classified initial presenting symptoms according to 1 of 14 symptom complexes (psychiatric/behavioural disturbance, cognitive impairment, motor/gait abnormalities, visual disturbances, headache, sleep disturbance, dizziness/vertigo, fatigue/malaise, sensory disturbance, speech disturbance, language disturbance, auditory disturbance, seizures and ‘other’). We evaluated presence of clinical features listed in the NCJDRSU structured questionnaire. We defined pyramidal signs as the presence of spasticity, spastic gait or pyramidal weakness; extrapyramidal signs as rigidity, dystonia or chorea; visual signs as hemianopia or cortical blindness; and cerebellar signs as cerebellar limb ataxia or gait ataxia.

### Diagnostic investigations

MRI studies were performed at referring sites across the UK. A NCJDRSU neuroradiologist reviewed each MRI using a standardised approach for the presence of cortical, basal ganglia and thalamic signal abnormalities on fluid-attenuated inversion recovery and diffusion-weighted imaging sequences, blinded to clinical information. Each study was graded as positive or negative (including suspicious but not meeting diagnostic criteria) based off the diagnostic criteria at the time of review. CSF sampling was undertaken locally, including routine biochemical and microbiological analyses. Frozen samples were provided to the NCJDRSU CSF laboratory for 14-3-3 and RT-QuIC analyses. Despite introduction into 2017 diagnostic criteria, our unit has analysed RT-QuIC since 2011. EEGs were undertaken in referring centres. Studies with periodic sharp wave complexes were graded positive.

We received informed consent for *PRNP* genetic sequencing. *PRNP* sequencing and codon 129 genotyping were conducted on blood or post-mortem brain material. Individuals with pathogenic *PRNP* mutations confirming an inherited prion disease were not included. Codon 129 genotyping was recorded as methionine homozygous (MM), valine homozygous (VV) or methionine-valine heterozygous (MV).

Post-mortem tissue was retrieved from referring centres. The tissue was screened for the presence of prion, amyloid-β, tau, α-synuclein and TDP43 pathology. Frozen tissue underwent biochemical analysis to identify the prion protein isotype. This was reported as type 1A, type 2A or mixed. The codon 129 genotype and prion protein isotype were combined to provide the molecular classification of the disease.

### Statistical analysis

We used IBM SPSS Statistics for Windows (version 27, Armonk, NY, USA) [21]. Statistical significance was defined as a *P*-value <0.05. Missing data were omitted from analysis. Continuous data were assessed for normality. Normally distributed continuous data are presented as mean (±SD). Non-normal continuous data are presented as median (interquartile range (IQR)). Categorical data were presented as frequency (percentage). Comparisons were made between age groups. Categorical data were analysed using Chi-square test. Time-to-event data underwent Kaplan–Meier survival analysis and log-rank testing. For calculation of diagnostic sensitivity of individual investigations, equivocal results were considered as negative.

## Results

### Baseline characteristics for total cohort

During the 10-year study period, there were 1,196 consecutive cases of sCJD. Of these, 21 cases were re-classified upon applying current diagnostic criteria to referrals prior to 2017. The study cohort characteristics are summarised in Table 1**.**

**Table 1:** Characteristics of study cohort (*n* = 1,196)

Feature	Statistic
Age of onset (years)	
Mean (±SD)	68.7 (±9.2)
Median (IQR)	68.9 (IQR: 63.0–74.9)
Range	25–94
Sex, *n* (%)	
Male	599 (50.1%)
Female	597 (49.9%)
Duration (months)	
Median disease duration (IQR)	4.1 (2.4–8.4)
Median time-to-diagnosis (IQR)	3.7 (2.1–7.1)
Diagnosis, *n* (%)	
In life	1011 (87.4%)
After death	146 (12.6%)
Diagnostic classification, *n* (%)	
Definite	395 (33.0%)
Probable	801 (67.0%)
Referral source, *n* (%)	
Neurologist	777 (65.0%)
Other physician	329 (27.5%)
Psychiatrist	8 (0.7%)
Pathologist	36 (3.0%)
Death certificate	4 (0.3%)
Other	42 (3.5%)
Investigation results, *n* (%)	
CSF	
14-3-3	
Positive	602 (65.8%)
Negative	313 (34.2%)
RT-QuIC	
Positive	848 (92.1%)
Negative	73 (7.9%)
MRI brain	
Positive	807 (89.2%)
Negative	28 (3.1%)
Suspicious (but not meeting diagnostic criteria)	70 (7.7%)
EEG	
Positive	156 (27.2%)
Negative	417 (72.8%)
Codon 129 polymorphism	
MM	437 (58.1%)
MV	169 (22.5%)
VV	146 (19.4%)
Prion protein isotype	
Type 1A	186 (60.6%)
Type 2A	100 (32.6%)
Mixed/other	21 (6.8%)

Abbreviations. CSF, cerebrospinal fluid; EEG, electroencephalography; IQR, interquartile range; MM, methionine homozygous; MRI, magnetic resonance imaging; MV, methionine-valine heterozygous; RT-QuIC, real-time quaking-induced conversion; SD, standard deviation; VV, valine homozygous.

For the total cohort, mean age of onset is 68.7 (±9.2) years. An age of onset histogram is demonstrated in Appendix 1. The prevalence is approximately equal between sexes. There were 395 (32.9%) neuropathologically confirmed diagnoses. Median time-to-diagnosis was 3.7 months (IQR: 2.1–7.1). Median duration of illness was 4.1 months (IQR: 2.4–8.4).

### Baseline characteristics for the over 80

There were 123 individuals (10.3%) with an age of onset >80 years and 1,063 individuals (88.9%) under 80. Age of onset was missing in 10 individuals (0.8%). The cohort characteristics by age group are summarised in Table 2.

**Table 2:** Characteristics of individuals with sCJD according to age of onset

	Under 80 years	Over 80 years	*P*-value
Number	1,063	123	
Female, *n* (%)	536 (50.4%)	55 (44.7%)	0.23
Neuropathological confirmation, *n* (%)	347 (32.6%)	44 (35.8%)	0.49
Median age at onset, years (range)	68.0 (25–79)	83.7 (80–94)	
Referral source, *n* (%)			
Neurologist	722 (67.9%)	51 (41.5%)	**<0.001**
Other physician	265 (24.9%)	62 (50.4%)	
Median disease duration, months	4.3 (IQR: 2.6–9.0)	3.2 (IQR: 2.0–6.2)	**<0.001**
Median time-to-diagnosis, months	3.7 (IQR: 2.1–7.3)	3.2 (IQR: 1.8–5.7)	**0.02**
Investigation sensitivity*			
RT-QuIC	91.9% (764/831)	92.9% (79/85)	0.74
14-3-3	65.8% (542/824)	67.1% (57/85)	0.81
MRI brain	91.4% (744/814)	67.8% (59/87)	**<0.001**
EEG	25.4% (129/508)	41.5% (27/65)	**0.006**
Codon 129			
MM	56.9% (387/680)	68.1% (47/69)	0.18
MV	22.9% (156/680)	18.8% (13/69)	
VV	20.1% (137/680)	13.0% (9/69)	
Prion protein isotype			
Type 1A	58.6% (160/273)	75.8% (25/33)	0.08
Type 2A	34.8% (95/273)	15.2% (5/33)	
Mixed/other	6.6% (18/273)	9.1% (3/33)	

Abbreviations. EEG, electroencephalography; MRI, magnetic resonance imaging; RT-QuIC, real-time quaking-induced conversion.

^*^Sensitivity defined as the proportion of positive results out of all tested individuals. Bold values denote statistical significance at the P < 0.05 level.

Referral to the NCJDRSU by a neurologist was less likely for those >80 years (41.5% vs 67.9%; *P* < 0.001). For late-onset CJD, 4.9% (6/123) were not referred during life, and only after post-mortem diagnosis.

### Disease duration

**Figure 1:**
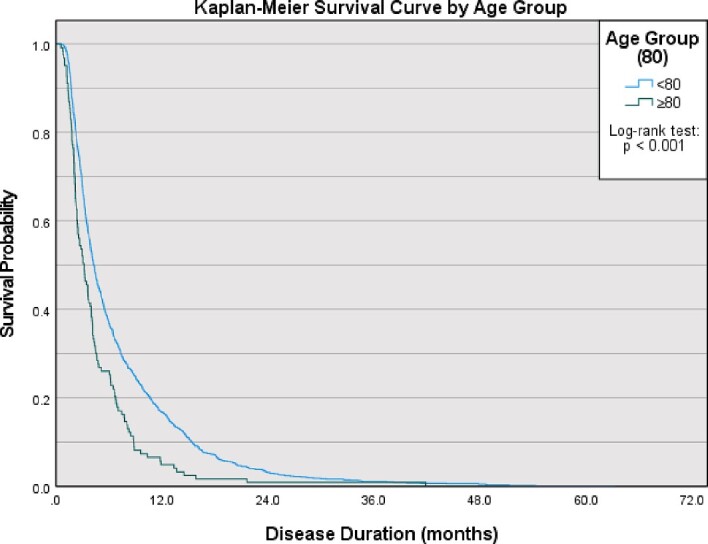
Kaplan–Meier survival curve by age group. Survival is significantly shorter in the over 80 (*P* < 0.001; log-rank test)

Disease duration data were present for 98.6% of the study population. The median disease duration was 3.2 months (IQR: 2.0–6.2) for the over 80, compared to 4.3 months (IQR: 2.6–9.0) in the under 80. Survival was shorter in the over 80 (*P* < 0.001, log-rank test) (Figure 1).

### Time-to-diagnosis

Time-to-diagnosis data were present in 96.3% of the study population. Median time-to-diagnosis was 3.2 months (IQR: 1.8–5.7) for the over 80, compared to 3.7 months (IQR: 2.1–7.3) in the under 80. Time-to-diagnosis was shorter in late-onset sCJD (*P* = 0.013, log-rank test).

### Presenting symptom

Presenting symptoms in the over 80 included cognitive impairment (48/123; 39.0%), motor/gait abnormality (27/123; 21.2%), psychiatric/behavioural disturbance (15/123; 12.2%), visual disturbance (9/123; 7.3%), speech disturbance (6/123; 4.9%), sleep disturbance (4/123; 3.3%), sensory disturbance (3/123; 2.4%), language disturbance (2/123; 1.6%), dizziness/vertigo (2/123; 1.6%), fatigue/malaise (2/123; 1.6%), auditory disturbance (1/123; 0.8%), headache (0/123; 0%), seizures (0/123; 0%) and other (1/123; 0.8%).

### Clinical features

In late-onset sCJD, cognitive impairment was observed in 97/97 (100%), psychiatric symptoms in 25/95 (26.3%), pyramidal signs in 46/89 (51.6%), extrapyramidal signs in 64/95 (67.4%), visual signs in 29/85 (34.1%), cerebellar signs in 53/81 (65.4%), myoclonus in 88/97 (90.7%) and akinetic mutism in 49/89 (55.1%). Seizures were identified in 11/94 (10.8%); sensory signs in 3/70 (4.3%). Lower motor neuron features were not seen in any individuals.

**Figure 2:**
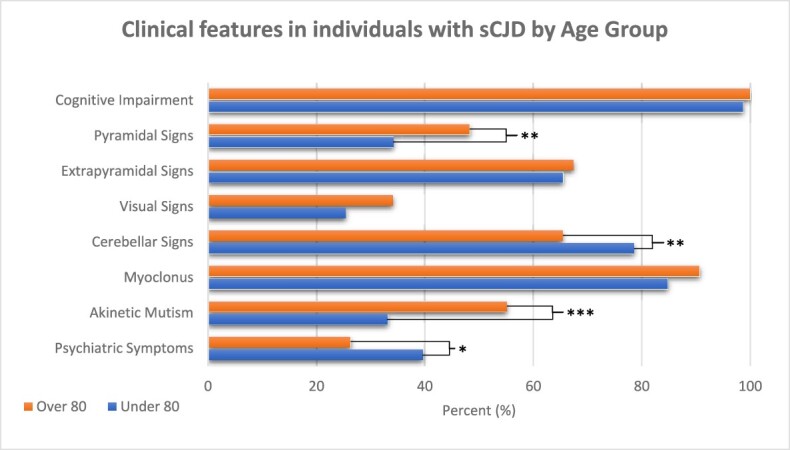
Clinical features in sporadic CJD by age group. Shown according to the frequency with which they were identified during the disease course up until the point of clinical assessment by NCJDRSU. Significance levels: *P < 0.05, **P < 0.01, ***P < 0.001

We compared clinical features between age groups (Figure 2). Pyramidal signs (48.3% vs 34.2%; *P* = 0.008) and akinetic mutism (55.1% vs 33.2%, *P* < 0.001) were more frequent in sCJD over 80. In contrast, psychiatric symptoms (26.3% vs 39.6%, *P* = 0.01) and cerebellar signs (65.4% vs 78.6%, *P* = 0.007) were less frequent. Between age groups, cognitive impairment (*P* = 0.27), myoclonus (*P* = 0.12) and extrapyramidal (*P* = 0.74) were comparable. In the over 80, there was a non-significant trend for more frequent visual signs (*P* = 0.08).

### Investigation profile

CSF RT-QuIC (69.1% vs 78.2%; *P* = 0.02) and CSF 14-3-3 (69.1% vs 77.5%; *P* = 0.04) were tested less frequently in late-onset sCJD. Between age groups, the testing frequency was similar for MRI brain (70.7% vs 76.6%; *P* = 0.15) and EEG (52.8% vs 47.8%; *P* = 0.29). Between older and younger age groups, the diagnostic sensitivity does not differ for CSF RT-QuIC (79/85; 92.9% vs 764/831; 91.9%) [*P* = 0.74] or CSF 14-3-3 (57/85; 67.1% vs 542/824; 65.8%) [*P* = 0.81]. EEG has a higher sensitivity in the over 80 (27/65; 41.5% vs 129/508; 25.4%) [*P* = 0.006]. MRI brain had inferior diagnostic sensitivity in the over 80 (59/87; 67.8% vs 744/814; 91.4%) [*P* < 0.001]. In the over 80, 21.8% (19/87) had suspicious but non-diagnostic imaging. The remaining 10.3% (9/87) had clearly negative imaging. In the under 80, imaging was suspicious in 6.3% (51/814) and negative in 2.3% (19/814).

### *PRNP* genetic sequencing and codon 129 genotyping

For *PRNP* genetic sequencing, there was less testing in the over 80 (43/123; 35.0% vs 527/1063; 49.6%) [*P* = 0.002]. The rate of codon 129 genotyping was similar between age groups (69/123; 56.9% vs 680/1063; 64.0%) [*P* = 0.09]. There was no difference in genotype distributions; MM (68.1% vs 56.9%), MV (18.8% vs 22.9%) and VV (13.0% vs 20.1%) [*P* = 0.18].

### Molecular classification

Molecular classification was possible in 77.7% (307/395) of neuropathologically confirmed cases. Between age groups, the proportion of molecular classification did not differ (33/44; 75% vs 273/347; 78.9%) [*P* = 0.58]. Late-onset sCJD has a non-significant trend towards type 1A isotype: type 1A (75.8% vs 58.6%), type 2A (15.2% vs 34.8%) and mixed/other (9.1% vs 6.6%) [*P* = 0.08]. Sample size limits statistical comparison by molecular classification. In the over 80, there is a greater proportion of MM1 cases (24/33, 72.7% vs 140/273, 51.3%) [*P* = 0.02]. Sample size limits further statistical comparison by molecular classification (Table 3).

**Table 3:** Molecular classification

	MM1	MM2	MV1	MV2	VV1	VV2	Other
Total, *n* (%)	165^*^ (53.7%)	19 (6.2%)	13 (4.2%)	32 (10.4%)	8 (2.6%)	49 (16.0%)	21 (6.8%)
Distribution by age group							
Under 80, *n* (%)	140 (51.3%)	18 (6.6%)	12 (4.4%)	31 (11.4%)	8 (2.9%)	46 (16.8%)	18^**^ (6.6%)
Over 80, *n* (%)	24 (72.7%)	1 (3.0%)	1 (3.0%)	1 (3.0%)	0 (0%)	3 (9.1%)	3^***^ (9.1%)
Median age (years)	70	66	67	67	57	71	71
Median disease duration (months)	3.0	11.8	4.0	15.7	14.5	5.6	7.6
Median time-to-diagnosis (months)	3.0	12.6	3.5	11.6	10.1	5.8	7.3
EEG sensitivity	32.8% (22/67)	0% (0/5)	30% (3/10)	0% (0/9)	0% (0/3)	0% (0/21)	
MRI sensitivity	88.5% (108/122)	100% (13/13)	75% (6/8)	91.7% (22/24)	100% (7/7)	88.2% (30/34)	
RT-QuIC sensitivity	98.2% (108/110)	54.5% (6/11)	80% (8/10)	90.5% (19/21)	0% (0/5)	90.6% (29/32)	
14-3-3 sensitivity	77.9% (88/113)	36.4% (4/11)	50% (5/10)	25% (5/20)	83.3% (5/6)	90.9% (30/33)	

Abbreviations. EEG, electroencephalography; MRI, magnetic resonance imaging; MM, methionine homozygous; MV, methionine-valine heterozygous; RT-QuIC, real-time quaking-induced conversion; VV, valine-valine homozygous.

^*^Age of onset not available for one MM1 case.

^**^MM1 + 2: 11 cases. MV1 + 2: 3 cases. VV1 + 2: 3 cases. MV with abnormal isotype: 1 case.

^***^MM1 + 2: 3 cases.

### Neuropathological profile

The neuropathological confirmation rate did not differ between age groups (44/123; 35.8% vs 347/1063; 32.6%) [*P* = 0.49]. In the over 80 group, co-pathology with amyloid-β, tau and vascular pathology are common. Extensive Lewy pathology was seen in one individual and mild Lewy pathology in two individuals. There was no TDP43 pathology. The neuropathological profile is demonstrated in Appendix 2.

## Discussion

The mean age of onset (68 years) is comparable to the literature [5, 12, 22–24]. Multiple national centres have observed increasing age over time [5, 12]. Despite high case-ascertainment surveillance, the age-specific incidence remains lower in the over 80 population [5]. We assessed the clinical characteristics and investigation profile of late-onset sCJD in a 10-year cohort of consecutive cases.

Late-onset sCJD has previously been described by national surveillance units [12, 25–27]. In our study and others, the over 80 have shorter survival and time-to-diagnosis [12, 26, 27]. Independent of age group, cognitive impairment and myoclonus are the dominant clinical features. In the over 80, cognitive impairment is the most frequent initial manifestation. Pyramidal signs are more common, in keeping with existing literature [26]. Psychiatric disturbance and cerebellar dysfunction are less common, in keeping with findings described in young-onset CJD [19, 28]. The frequency of extrapyramidal signs is similar between ages, and there is a non-significant trend towards more visual signs in the over 80. In a German surveillance study, both were less prevalent in older adults [26]. Despite shorter time-to-diagnosis, our study demonstrates that older adults are clinically advanced with a higher proportion of akinetic mutism at diagnosis.

We observed similar rates of investigation with MRI and EEG in both groups, but CSF biomarkers are less frequently tested. CSF RT-QuIC is the most sensitive (92%) and specific (100%) ante-mortem investigation [8]. We are the first to show the comparable sensitivity in the over 80 population. MRI brain is less sensitive in late-onset sCJD, which is reflected in previous data [12, 26]. Despite this, 68% of negative scans were suspicious but non-diagnostic. In practice, we recommend MRI brain in the routine work-up as MRI features are diagnostic or suspicious in 90%. We have shown that EEG is more sensitive in the over 80, although the evidence from surveillance studies is inconsistent [26, 27]. Our EEG findings are in keeping with the wider literature, as typical EEG findings are associated with both shorter disease duration and increasing age [29]. In addition, EEG is highly sensitive in MM1 cases [29], which we have seen most frequently in the over 80.

The biological basis for differences in clinical features with age may include underlying pathological subtypes as defined by the Parchi system [3]. In older individuals with sCJD, the dominant subtypes are MM1 and MV1, associated with frequent pyramidal features and rapid progression; in contrast, younger individuals have higher rates of VV2 and MV2 subtypes, featuring ataxic-predominant phenotypes and slower progression [30]. Our study has shown a greater proportion of MM1 in the over 80; however, sample size limits further statistical comparison.

Late-onset CJD was predominately referred by non-neurologists, which is in contrast to the majority of the remaining cohort being referred by neurologists. Pyramidal-predominant presentations may mimic stroke, with cases being assessed in stroke units that are largely led by non-neurologists in the UK. Likewise, older individuals with rapidly progressive cognitive and/or motor disorders may be assessed by geriatricians and psychiatrists with expertise in this domain. Older individuals may have frequent comorbidities compared to those in the typical age range, with the potential for misattribution. This was not assessed in this study. Finally, it is possible that older adults presenting with CJD-related features are less likely to be referred to or assessed by local neurologists for other reasons, and may undergo lower rates of admission to tertiary neurosciences units.

Our findings complement existing literature by clarifying the clinical features and investigation profile. This study adds the first evaluation of CSF RT-QuIC in older adults since the implementation of the 2017 diagnostic criteria. In addition, we have demonstrated the neuropathological profile in 44 late-onset sCJD cases. This study benefits from comprehensive prospective data capture using a population-based approach. This 10-year cohort is attributed to a national surveillance system with a high rate of case ascertainment.

Our main limitation relates to missing pathological and genetic data. Across the study period, the post-mortem rate has fallen steeply from ~50 to 10%, which is attributable to improved diagnostics in life [5]. *PRNP* genetic sequencing is incomplete, which assumes the risk that genetic aetiologies may be undiscovered in the untested population. These are representative of using a real-world population. The clinical features captured are limited by the time point of clinical review. The clinical features may be influenced by illness severity; however, retrospective capture through clinical history and review of notes provides robustness.

Future work may consider the use of the Medical Research Council Prion Disease Rating Scale to record the degree of disease severity [31]. Larger neuropathological case series in highly ascertained populations with molecular phenotyping would be beneficial to confirm the trend towards MM1 in very old people to establish if this difference is due to ascertainment or underlying biology.

Geriatricians should suspect sCJD in rapidly progressive neurodegenerative syndromes. Furthermore, stroke physicians may consider sCJD in atypical stroke-like presentations in view of the frequent pyramidal signs. In contrast to patients with stroke, in which deficits are sudden-onset and maximal at onset, sCJD causes relentless deterioration and emergence of new deficits.

We emphasise to geriatricians that CSF RT-QuIC has been underutilised, and that MRI alone may not identify cases as readily due to its lower sensitivity. CSF RT-QuIC proves to be highly sensitive across all age groups. Geriatricians should consider the combination of MRI and CSF RT-QuIC in suspected CJD, alongside discussion with a specialist prion unit. Diagnosis is important to confirming terminal illness and excluding reversible differentials; the combination of MRI and CSF RT-QuIC readily differentiates sCJD from its mimics [32]. There are currently no disease-modifying therapies for sCJD, but early diagnosis facilitates the preparation of family members, access to supportive care and end-of-life care planning. Aggressive illness and low index of suspicion may be a barrier to ante-mortem diagnostic investigations. Post-mortem is valuable where diagnosis is not available in life.

## Supplementary Material

aa-23-1578-File002_afae086
