# Supplementary material for: Sporadic Creutzfeldt-Jakob disease in adults over 80 years: a 10-year review of United Kingdom surveillance
Source: Age Ageing. 2024 May 5;53(5):afae086. doi: 10.1093/ageing/afae086 (PMC11070723; doi:10.1093/ageing/afae086)
Supplement: aa-23-1578-File002_afae086 [file aa-23-1578-file002_afae086.docx]

# **Sporadic Creutzfeldt-Jakob disease in adults over 80 years: A 10-year review of United Kingdom surveillance.**

## Appendix 1


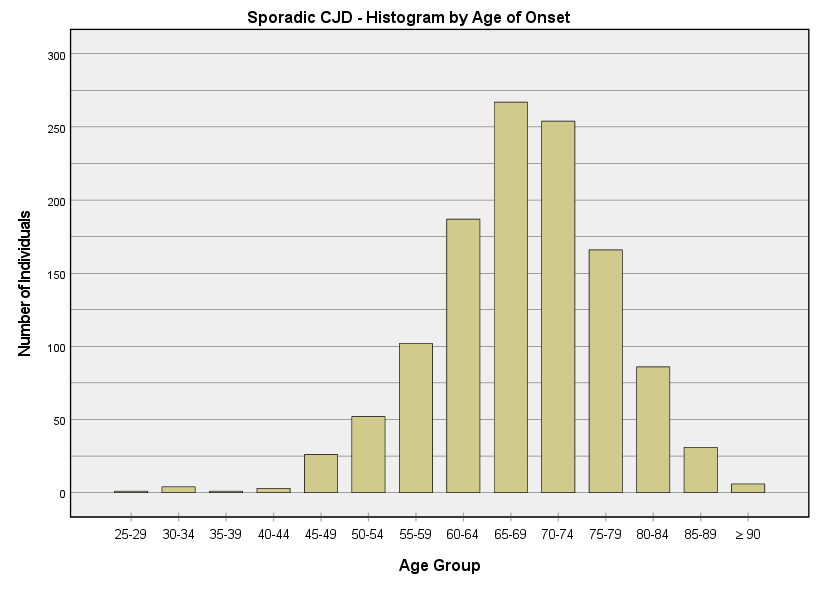


**Appendix 1**: Individuals with sporadic CJD according to age of onset (years). Histogram demonstrating frequencies of individuals with sCJD according to defined age categories. The most frequent age of onset was 65-69 years.

## Appendix 2

| **Appendix 2. Neuropathological Profile in the Over 80** | | | | | | | |
| --- | --- | --- | --- | --- | --- | --- | --- |
| Patient | Molecular classification | Aβ pathology | Tau Pathology | α-syn pathology | TDP43 | Arteriosclerosis | CAA |
| 1 | MM1 | - | - | - | - | - | - |
| 2 | MM1 | None/mild |  | None | - | Mild/NOS | - |
| 3 | n/a | - | - | - | - | - | - |
| 4 | MM2 | Mod/sev | None/mild | None | None | - | - |
| 5 | VV2 | None/mild | None/mild | None | - | Mod/sev | - |
| 6 | VV2 | None/mild | None/mild | None | - | Mild/NOS | - |
| 7 | MM1 | None/mild | None/mild | None | None | Mod/sev | Mod/sev |
| 8 | MM1 | None/mild | None/mild | None | - | Mild/NOS | - |
| 9 | MM1 | None/mild | None/mild | None | - | Mild/NOS | - |
| 10 | VV (?) | Mod/sev | None/mild | None | None | Mild/NOS | Mod/sev |
| 11 | MM1 | None/mild | None/mild | None | - | Mild/NOS | - |
| 12 | n/a | None/mild | None/mild | Mild | - | Mild/NOS | - |
| 13 | MM1 | Mod/sev | Mod/sev | None | None | Mod/sev | Mod/sev |
| 14 | MV | None/mild | Mod/sev | None | None | Mild/NOS | Mild/NOS |
| 15 | MM1 | Mod/sev | Mod/sev | None | None | Mod/sev | Mild/NOS |
| 16 | n/a | Mod/sev | Mod/sev | - | - | - | - |
| 17 | MM1 | None/mild | None/mild | None | - | Mod/sev | - |
| 18 | MM1 | None/mild | None/mild | None | None | Mild/NOS | None |
| 19 | MM1 | Mod/sev | None/mild | None | None | Mild/NOS | Mod/sev |
| 20 | MM1 | None/mild | Mod/sev | None | None | - | None |
| 21 | MM1 | Mod/sev | None/mild | Mild | None | None | Mod/sev |
| 22 | MM1+2 | None/mild | None/mild | None | - | Mod/sev | - |
| 23 | MM1 | None/mild | None/mild | None | - | Mod/sev | - |
| 24 | MM1 | - | - | - | - | - | - |
| 25 | MM1 | None/mild | None/mild | None | - | Mod/sev | - |
| 26 | MM1 | None/mild | None/mild | None | - | Mod/sev | - |
| 27 | MV2 | None/mild | None/mild | None | - | Mild/NOS | - |
| 28 | MM1 | None/mild | Mod/sev | None | - | Mod/sev | Mod/sev |
| 29 | VV (?) | None/mild | None/mild | None | None | Mod/sev | None |
| 30 | MM1 | None/mild | None/mild | None | - | Mod/sev | - |
| 31 | MM1 | None/mild | None/mild | None | - | Mod/sev | None |
| 32 | MM1+2 | - | - | - | - | - | - |
| 33 | MV1 | Mod/sev | None/mild | None | - | - | - |
| 34 | VV2 | Mod/sev | Mod/sev | None | None | - | Mod/sev |
| 35 | n/a | Mod/sev | None/mild | Mod/sev | - | - | Mild/NOS |
| 36 | MM1 | Mod/sev | Mod/sev | None | None | Mild/NOS | Mild/NOS |
| 37 | MM1 | Mod/sev | Mod/sev | None | - | - | Mod/sev |
| 38 | MM (?) | None/mild | None/mild | None | None | Mild/NOS | - |
| 39 | MM1+2 | Mod/sev | Mod/sev | None | None | - | None |
| 40 | MM1 | Mod/sev | None/mild | None | None | - | Mod/sev |
| 41 | n/a | None/mild | None/mild | None | None | Mod/sev | - |
| 42 | MM1 | None/mild | None/mild | None | None | - | - |
| 43 | n/a | - | - | - | - | - | - |
| 44 | n/a | Mod/sev | Mod/sev | None | - | None | Mild/NOS |
| **Abbreviations.** Aβ, Amyloid beta. α-syn, alpha synuclein. CAA, cerebral amyloid angiopathy. MM, methionine homozygous. MV, methionine-valine heterozygous. NOS, not otherwise specified. TDP43, TAR DNA-binding protein 42. VV, valine-valine homozygous. | | | | | | | |
